# Determinants of blood glucose control among people with Type 2 diabetes in a regional hospital in Ghana

**DOI:** 10.1371/journal.pone.0261455

**Published:** 2021-12-22

**Authors:** Sampson Kafui Djonor, Ignatius Terence Ako-Nnubeng, Ewurama Ampadu Owusu, Kwadwo Owusu Akuffo, Pricillia Nortey, Eldad Agyei-Manu, Anthony Danso-Appiah

**Affiliations:** 1 Department of Epidemiology and Disease Control, School of Public Health, College of Health Sciences, University of Ghana, Legon, Ghana; 2 Central Laboratory, Korle-Bu Teaching Hospital, Korle-Bu, Accra, Ghana; 3 Department of Medical Laboratory Sciences, School of Biomedical and Allied Health Sciences, College of Health Sciences, University of Ghana, Legon, Ghana; 4 Department of Optometry and Visual Science, College of Science, Kwame Nkrumah University of Science and Technology, Kumasi, Ghana; 5 Centre for Evidence Synthesis and Policy, School of Public Health, University of Ghana, Legon, Accra, Ghana; Tufts Medical Center Molecular Cardiology Research Institute, UNITED STATES

## Abstract

**Aims:**

To assess the determinants of glycaemic control among patients with Type 2 diabetes mellitus (T2DM) presenting at the Greater Accra Regional Hospital, Ghana.

**Methods:**

The study employed semi-structured questionnaires and review of clinical records of patients 16 years and above with Type 2 Diabetes.

**Results:**

The mean age of participants was 56.6 ± 13.8 years, with majority (71.6%) being females. A total of 161 (59.4%) of patients had poor glycaemic control (HbA1c ≥8.1%; 95% CI: 53.6 to 65.3%). Poor glycaemic control was significantly associated with high body mass index of the patient (AOR 13.22; 95% CI: 1.95 to 89.80), having only elementary education (AOR 5.22, 95% CI 2.12–12.86, p<0.0001) and being on insulin therapy (AOR 2.88; 95% CI: 1.05 to 7.88). On the other hand, seldom coffee intake (AOR: 0.27; 95% CI: 0.11 to 0.64), high physical activity (AOR 1.57, 95% CI 1.06–2.35, p = 0.025) and having a cardiovascular disease (AOR: 0.15; 95% CI: 0.05 to 0.46) appeared to positively influence glycaemic control. Self-monitoring of blood glucose and diet interventions did not appear to influence glycaemic control.

**Conclusions:**

The study results showing that a high proportion of patients attending the Diabetes Clinic with uncontrolled diabetes has serious implications for the management of T2DM diabetes as it suggests that current hospital-based treatment measures are less effective. Comprehensive management of T2DM targeting all the key factors identified in this study and incorporating a multispectral collaborative effort based on holistic approach, combined with non-pharmacological components are strongly warranted.

## Introduction

Type II Diabetes Mellitus (T2DM), one of the four major types of diabetes mellitus (DM) [1], is a leading cause of ill-health that requires continuous medical care, ongoing self-management, and support to prevent or reduce complications from hyperglycemia, hypoglycemia, hypertension, cardiovascular diseases, or kidney failure [2]. Approximately 90% of all diabetes cases are T2DM. The International Diabetes Federation (IDF) in 2019 estimated the global prevalence of diabetes to be 9.3% (translating to 713.9 million with diabetes) and projected this to rise to 10.2% and 10.9% by 2030 and 2045, respectively [3].

Several methods have been proposed for the diagnosis and monitoring of DM with varying accuracies [1]. Glycated haemoglobin (HbA1c) and fasting plasma glucose (FPG) tests are considered more accurate and better, but these are slightly expensive and come with cost implications, especially for patients in low- and middle-income countries (LMICs) [4]. HbA1c is an effective biomarker of long-term glycaemic control, an efficient predictor of diabetes complications and independent risk factor for stroke and coronary heart disease, hence ideal for identifying and monitoring DM patients who are at high risk of cardiovascular complications [5]. Although little controversy surrounds the ideal target for blood glucose control for those with diabetes on a range of 6.0–7.5% HbA1c, the American Diabetes Association (ADA) and the European Association for the Study of Diabetes (EASD) recommend HbA1c cut-off point of 7.0% (53.0mmol/mol) as optimal [6, 7]. In Ghana, however, most HbA1c assay results from reputable laboratories such as Synlab Ghana Limited, MDS Lancet Laboratories Limited and Greater Accra Regional Hospital laboratory, which work hand-in-hand with health facilities and clinicians come with a glycaemic control guideline (reference range) that interprets or classifies diabetes status as HbA1c <6.0% to be non-diabetes; 6.0%–8.0% as good diabetes control; 8.1%–10.0% as fair diabetes control, and >10.0 as poor diabetes control [8].

Regardless of the age of the patient, T2DM is difficult to manage or control as it demands on strict adherence to health education protocols [9]. Non-pharmacological or conventional treatment of T2DM involves the use of dietary interventions and physical activity [10], whereas pharmacological treatment consists of the use of drugs such as sulfonylurea (in normal-weight individuals) and metformin (in overweight and obese patients), or insulin when diabetes is first detected and the blood glucose is very high, or when a patient’s blood glucose levels remain persistently high [11]. In most T2DM control therapies, individuals are put on more than a single medication, and management involves combination therapy of pharmacological and non-pharmacological interventions.

An essential component to healthy living and longevity in T2DM affected individuals is to have adequate glycaemic regulation [12]. Uncontrolled diabetes has dire consequences on well-being and health. Therefore, intensive glycaemic control prevents or reduces the incidence of micro-and macro-vascular complications [13]. Increased morbidity and mortality are associated with hyperglycemia among in- and out-patients [14], and as such, plasma glucose and HbA1c should be intensively controlled to desired levels to reduce T2DM-related complications and mortality. The Standards of Care of Diabetes [15] indicates that educating a person with diabetes, together with his/her family, is the cornerstone of good diabetes management. However, some T2DM patients on treatment hardly make progress in their blood glucose control for unknown reasons even with adequate knowledge and support of their families.

A body of evidence suggests an association between body mass index (BMI), physical inactivity, formal education and diet on uncontrolled blood glucose among T2DM [16]. However, there is paucity of data on factors influencing blood glucose control in Ghana’s T2DM patients. This has led to the Ghana Health Service calling for urgent research in this area to provide evidence to support sound policies and management T2DM [17], particularly in the Greater Accra Region recording the highest number of DM cases (37,057 as compared to 1,209 cases in the Upper West Region) and with 8 cases per any 1000 OPD cases in the region for year 2017 [18]. The country’s DM prevalence still remains high (6.5%) [19]. This study was conducted as a direct response to demand, policy, and context-specific research calls to assess determinants of blood glucose control among people with T2DM. The study placed emphasis on optimal blood glucose control and analyzed key factors that could influence glycaemic control. Self-monitoring blood glucose (SMBG), despite its benefits and value for glycaemic control, is less commonly used in LMICs. This study also investigated SMBG and its potential for blood glucose control in T2DM in Ghana.

## Materials and methods

### Study design and site

This prospective cross-sectional study was conducted from May to June, 2018, and used both questionnaire and review of clinical records of patients with T2DM attending the Diabetic Clinic or the Medical Ward of the Greater Accra Regional Hospital, an ultra-modern 620-bed capacity facility in the Accra Metropolis. The hospital provides expanded access and quality healthcare to over six million people across the Greater Accra Region, and also serves as one of the major referral hospitals in Ghana.

### Study population

Patients diagnosed with T2DM and presenting at the Diabetes Clinic or on admission at the medical ward of the Greater Accra Regional Hospital participated in this study. Diagnostic criteria involved using consistently high FPG results greater than or equal to 126 mg/dL [7.0 mmol/L] and random plasma glucose [RPG] results greater than or equal to 200 mg/dL [11.1 mmol/L]) and confirmed by HbA1c. Only participants whose HbA1c results were recorded not later than three months and who had been on treatment for at least six months, were included. T2DM patients with HbA1c records but who refused consent were excluded.

### Sample size determination and sampling

A sample size of 270 was determined from Fisher’s formula [*N = z*^*2*^ *(pq)/d*^*2*^] at confidence level of 95% (1.96), estimated proportion (p) of participants with poor glycaemic control of 20% from an earlier study [20], margin of error (d) = 0.05 and 10% non-response rate. A consecutive sampling method was employed in this study where respondents who fell within the inclusion criteria were enrolled until the estimated minimum sample size was achieved. Overall, 271 patients were included in the study. S1 Fig is a flowchart summarizing participants’ enrolment, eligibility and number included in the analyses.

### Data collection

A semi-structured questionnaire was used to collect data. The Short Last 7 Days Self-Administered International Physical Activity Questionnaire (IPAQ), 24hr Dietary Recall and the Food Frequency Questionnaire (FFQ) were adapted and used to collect data. Among others, the questionnaires assessed patients’ socio-economic status, how much involved they were in the treatment of their diabetes, dietary pattern, physical activity, and factors affecting their glycaemic control. The results of HbA1c test and current BMI were obtained from patients’ medical records. The questionnaires were pretested and adapted for use. Three research assistants were trained on appropriate data collection and entry to ensure uniformity and accuracy of data. Data collection started from May to June 2018.

#### Physical activity

The IPAQ questionnaire, with scoring done using generated spreadsheets [21] allowed participants’ level of physical activity (PA) to be categorized into:

1. low PA (neither moderate nor vigorous activity in a week),
2. moderate PA (three or more days of vigorous activity ≥ 20 minutes per day; or ≥5 days of moderate-intensity activity and/or walking of at least 30 minutes each day; or a combination of vigorous activity, or moderate-intensity activities that sum up to ≥ 600 metabolic equivalents (METs) minutes per week, or
3. high PA (20 minutes of vigorous activity for ≥3 days amounting to ≥1500 METs minutes per week or any combinations of vigorous activity, moderate-intensity activities or walking that results in a total of ≥3000 METs-minutes/week).

#### Dietary pattern

Using the FFQ, food intake was categorized on a Likert scale into more than once a day, once a day, 2 to 3 times per week, seldom and never. The 24Hr Dietary Recall was assessed using Samsung Health Application version 5.17.1.003 (Samsung Electronics Co. Limited, 2018). Quantities of food intake by participants were entered into the App, which generated their total energy intake in kilocalories (kcal), and fat, carbohydrate, protein, and fiber intake in grams (g). All parameters were converted to kcal (fibre, carbohydrate, and protein intake in grams were converted to kcal with a multiplication factor of 4 whereas the multiplication factor for fats was 9 and outcome expressed as percentages. Less than 20g/kcal of fibre was classified as low intake, 20 to 35g/kcal as moderate or normal intake, and > 35g/kcal was considered as high intake [22]. For fats, <20% kcal intake, intake between 20–35%, and > 35% intake were considered as low, moderate, and high intake respectively [23]. For proteins, <10% kcal, 10–20% kcal, and > 20% kcal intake were considered as low, moderate and high protein intake, respectively [24]. Less than 40% kcal, 40–60% kcal, and >60% kcal were considered as low, moderate and high carbohydrate intake, respectively [22].

### Data analysis

Data were analyzed using MS Excel 2013 and STATA version 15.0 (StataCorp LLC, College Station, TX 77845, USA). Descriptive statistics were used to determine the frequencies and percentages of demographic characteristics of the respondents. Chi-Square (X^2^) statistic was used to find baseline associations between glycaemic control and physical activity, dietary patterns, and sociodemographic characteristics. For significant associations, bivariate and multivariate logistic regression analyses were used to establish the strength of (true) associations between glycaemic control and independent variables of the study (whiles including all theoretically known variables that are associated with glycaemic control such aspirin intake, being on anti-retroviral drugs, erythropoietin, iron, and vitamin B_12_ supplements, smoking, alcohol intake, having splenectomy, recent transfusion, splenomegaly, arthritis, and sickle cell disease). A paired *t*-test was used to assess any significant improvement (change in HbA1c results) following treatment.

### Ethical considerations

The study adhered to good clinical practice guidelines and the tenets of the ethics Declaration of Helsinki. All participants gave written informed consent and for those who could not read or write, the study was explained to them in a language they understood (Ga, Ewe and Akan) after which they provided a thumb print before they could participate in the study. Written informed consent was obtained from parents or guardians of minors included in the study. Ethical approval was obtained from the Ghana Health Service Ethics and Review Committee (ID Number GHS-ERC: 157/12/17).

## Results

### Socio-demographic characteristics and glycaemic control

The mean age of participants recruited in this study (n = 271) was 56.6 ± 13.8 years (95% CI: 16 to 89 years), with the majority being females (71.59%; n = 194) (Table 1). Almost all the study participants lived in urban communities (98.89%; n = 268), with the rest being residents of peri-urban communities. Majority (83.03%; n = 225) of the participants were reportedly Christians. Over 70% of the respondents earned less than Ghȼ500.00 (~$84.75) monthly, and over 50% (n = 137) had low education, only up to elementary or Junior High School. Participants’ occupation generally cuts across borders; from managerial positions to agricultural, craft and related trades, etc.

**Table 1:** Glycaemic control, socio-demographic and economic characteristics of respondents.

Variables	Glycaemic control [n = 271]		X^2^	*p*-value
	Good [n = 110] (%)	Poor [n = 161] (%)		
**Age (as at last birthday) in years**			7.4070	0.264#
15–24	2 (1.8)	1 (0.6)		
25–29	3 (2.7)	2 (1.2)		
30–39	12 (10.9)	21 (13.0)		
40–49	12 (10.91)	24 (14.9)		
50–59	32 (29.1)	41 (25.5)		
60–69	24 (21.8)	49 (30.4)		
≥70	25 (22.7)	23 (14.3)		
**Gender**			0.0418	0.838
Male	32 (29.1)	45 (28.0)		
Female	78 (70.9)	116 (72.1)		
**Level of Education**			**13.958**	**0.003**
No formal education	9 (8.2)	15 (9.3)		
Elementary/JHS	34 (30.9)	79 (49.1)		
SHS Education	31 (28.2)	42 (26.1)		
Tertiary Education	36 (32.7)	25 (15.5)		
**Religion**			4.1852	0.161#
Christianity	97 (88.2)	128 (79.5)		
Islam	13 (11.8)	31 (19.3)		
Traditional	0 (0.0)	1 (0.6)		
Others	0 (0.0)	1 (0.6)		
**Occupation**			13.4656	0.066#
Managers	1 (0.9)	6 (3.7)		
Skilled professional	30 (27.3)	26 (16.2)		
Clerical Support	5 (4.6)	5 (3.1)		
Service and Sales	31 (28.2)	56 (34.8)		
Agricultural	3 (2.7)	0 (0.0)		
Craft & Related Trade	6 (5.5)	12 (7.5)		
Labourer	0 (0.0)	2 (1.2)		
Unemployed	34 (30.9)	54 (33.5)		
**Residence**			0.0663	1.000#
Urban	109 (99.1)	159 (98.8)		
Peri-Urban	1 (0.9)	2 (1.2)		
**Estimated Income Level** *			8.7056	0.108#
< Ghȼ200	33 (30.3)	66 (41.3)		
Ghȼ200—Ghȼ499	43 (39.5)	51 (31.9)		
Ghȼ500—Ghȼ999	16 (14.7)	26 (16.3)		
Ghȼ1000—Ghȼ1999	12 (11.0)	15 (9.4)		
Ghȼ2000—Ghȼ3999	5 (4.6)	1 (0.6)		
Ghȼ4,000 and Over	0 (0.0)	1 (0.6)		

^#^ Fisher’s exact p-value, all others are Chi-square (X^2^) p-values.

*1$ = Ghȼ5.90 as at 23^rd^ December, 2020.

Table percentages were arrived at using column totals.

The mean HbA1c was 8.65 ± 2.33% (95% CI: 4.1 to 16.2%). A total of 11.4% of participants had HbA1c value less than or equal to 6.0%, 18.82% had HbA1c values between 6.0 and 7.1%, whiles 69.74% had HbA1c values 7.1% and above. Concerning HbA1c categorization in this study, values of 8.0% and below were considered as good glycaemic control, whereas values above 8.0% were regarded as poor glycaemic control. On this basis, the proportion with poor glycaemic control among study participants was 59.41 ± 0.03% (95% CI: 53.6 to 65.3%).

HbA1c results of 190 participants at their earlier years of treatment (those with over 2 years of having T2DM) were assessed, and their mean HbA1c was 8.94 ± 2.37% (95% CI: 5.2 to 17.2%). A two-sample proportion test found that the participants’ HbA1c value was reduced at the time of this study compared to that at the beginning of treatment (mean reduction of 39.4 ± 1.7%; 95% CI: 14.9 to 63.8%; p = 0.002). The study found that only educational level was significantly associated with glycemic control. The higher one’s level of education, the lower the probability of that individual to having a higher HbA1c value. The study did not find that age, gender, religion, residence, occupation, or monthly income as significantly associated (Table 1).

### Glycaemic control among T2DM patients

Information was obtained on participants’ diabetes status, BMI, duration of living with T2DM condition, type of treatment therapy being used and effectiveness, for glycaemic control (Table 2). Only BMI (X^2^: 8.5165; p = 0.035) and being on insulin (X^2^:6.9507; p = 0.008) were found to be independently associated with glycaemic control.

**Table 2:** T2DM and interventions for glycaemic control in patients attending the Greater Accra Regional Hospital, Ghana.

Variables	Glycaemic control [n = 271]		X^2^	*p*-value
	Good [n = 110] (%)	Poor [n = 161] (%)		
**BMI (Kg/m** ^**2**^ **)** *			**8.5165**	**0.035** #
(Mean: 28.64±3.97, 95% CI 19.6–47.0 Kg/m^2^)				
18–24.9	26 (24.5)	26 (16.25)		
25.0–29.9	53 (50.0)	70 (43.5)		
30.0–34.9	24 (22.6)	50 (31.1)		
≥35.0	3 (2.8)	15 (9.3)		
**Duration of Disease**			7.2544	0.104#
Within a year	23 (20.9)	36 (22.4)		
2–5 years	51 (46.4)	59 (36.7)		
5–10 years	23 (20.9)	37 (23.0)		
10–20 years	13 (11.8)	21 (13.0)		
≥20 years	0 (0.0)	8 (5.0)		
**Type of Treatment**				
Diet	29 (26.4)	40 (25.5)	0.0794	0.778
Exercise	14 (12.7)	21 (13.0)	0.0058	0.939
Insulin	10 (9.1)	34 (21.1)	**6.9507**	**0.008**
Oral Medication	103 (93.6)	152 (94.4)	0.0704	0.791
**Idea on Improvement Status**			6.0138	0.051#
Improved Condition	98 (89.1)	124 (77.5)		
No Change in Condition	7 (6.4)	22 (13.8)		
Condition Worsened	5 (4.6	14 (8.8)		
**Physical Activity Level**			**6.4612**	**0.040**
High	11 (10.0)	13 (8.1)		
Moderate	38 (34.6)	35 (21.7)		
Low	61 (55.5)	113 (70.2)		
**Self-Monitoring Blood Glucose**			0.4300	0.512
Utilization	53 (49.1)	72 (45.0)		
Non-Use	55 (50.9)	88 (55.0)		

^#^Fisher’s exact p-value, all others are Chi-square (X^2^) p-values.

*BMI classification: normal (18.0–24.9), overweight (25.0–29.9), obese (30.0–34.9) and morbidly obese (≥35.0 Kg/m^2^).

Table percentages were arrived at using column totals.

### Physical activity and blood glucose control

The respondents’ physical activity level was classified as low, moderate or high, and its effect on glycemic control was assessed (Table 2). The majority of the study participants had a low level of physical activity (64.2%; n = 174); only a handful of respondents had high physical activity levels (8.9%; n = 24). Physical activity level was significantly associated with glycaemic control (X^2^: 6.6412; p = 0.040). More importantly, the level of physical activity was found to be associated with gender (X^2^: 11.6091; p = 0.003) as more males than females tended to do exercise. As such, controlling for age and gender, high physical activity was significantly associated with good glycaemic control (AOR 1.57, 95% CI 1.06–2.35, p = 0.025).

### Self-monitoring of blood glucose and glycaemic control

Participants were asked whether they had glucometer and hence, practised self-monitoring blood glucose (SMBG) therapy. In all, 143 out of 268 (53.36%) respondents gave an affirmative response to having a blood glucose monitor. Of those who did not use glucose monitors (n = 145), 32.4% (n = 47) stated financial constraints as their challenge. A total of 49.7% (n = 72) attributed their inability to use glucose monitors to being unaware of SMBG. Also, a total of 10.3% (n = 15) attributed it to challenges in testing and results interpretation, whiles the rest (7.6%) did not have any reason for not utilizing SMGB therapy. SMBG was not found to have a statistically significant association with glycaemic control (Table 2). However, its utilization was significantly associated with the duration of disease (X^2^:51.4884, p< 0.0001). A longer duration of T2DM condition corresponded to 2.4 times increased odds of having a blood glucose monitor (95% CI: 1.78 to 3.18; p<0.0001).

### Diet and blood glucose control

FFQ and 24Hr dietary recall were used to assess the level of intake of various foods by participants and their dietary patterns. With the exception of intake of fast foods, intake of coffee and salty snacks that were found to be significantly associated with glycaemic control (X^2^: 8.0146, p = 0.032; X^2^: 10.8889, p = 0.025; and X^2^: 8.2718, p = 0.027, respectively), all the other categories of foods did not show any significant association (Tables 3 and 4).

**Table 3:** Glycaemic control and food categories of 24hr dietary recall.

Food Group	Glycaemic control [n = 262]		X^2^	*p*-value
	Good [n = 107] (%)	Poor [n = 155] (%)		
**Percentage carbohydrate per kcal intake**			4.3069	0.119#
(Mean 63.76 ± 10.42; 95%CI 12.74 to 85.15)				
High Intake (>60%)	77 (72.0)	100 (64.5)		
Normal Intake (40–60%)	25 (23.4)	52 (33.6)		
Low Intake (<40%)	5 (4.7)	3 (1.9)		
**Percentage protein per kcal intake**			2.1870	0.335
(Mean 15.79 ± 5.37; 95% CI 1.72 to 31.43)				
High Intake (>20%)	21 (19.6)	35 (22.6)		
Normal Intake (10–20%)	75 (70.1)	96 (61.9)		
Low Intake (<10%)	11 (10.3)	24 (15.5)		
**Percentage fat per kcal intake**			0.9951	0.608
(Mean 20.45 ± 10.02; 95% CI 7.09 to 81.46)				
High Intake (>35%)	11 (10.3)	19 (12.3)		
Normal Intake (20–35%)	28 (26.2)	47 (30.3)		
Low Intake (<20%)	68 (63.6)	89 (57.4)		
**Fibre intake (g/kcal)**			2.5354	0.281
(Mean 28.45 ± 10.56; 95% CI 4.80 to 79.30)				
High Intake (>35g)	29 (27.1)	31 (20.0)		
Normal Intake (20 -35g)	62 (57.9)	92 (59.4)		
Low Intake (<20g)	16 (15.0)	32 (18.3)		

^#^Fisher’s exact p-value, all others are Chi square (X^2^) p-values.

Table percentages were arrived at using column totals.

**Table 4:** Glycaemic control and frequency of various food intake among people with T2DM.

Intervention (food intake)	Glycaemic control [n = 271]		X^2^	*p*-value (Fisher’s Exact)
	Good [n = 110] (%)	Poor [n = 161] (%)		
**Milk**			4.2246	0.365
Never	11 (10.0)	26 (16.2)		
Seldom	38 (34.6)	60 (37.3)		
2–3 times/week	41 (37.3)	56 (34.8)		
Once/day	18 (16.4)	18 (11.2)		
>Once/day	2 (1.8)	1 (0.6)		
**Sweets** **			3.5863	0.263
Never	46 (41.8)	50 (31.1)		
Seldom	54 (49.1)	92 (57.1)		
2–3 times/week	9 (8.2)	18 (11.2)		
Once/day	1 (0.9)	1 (0.6)		
**Meat and poultry**			2.4800	0.634
Never	24 (21.8)	27 (16.8)		
Seldom	34 (30.9)	56 (34.8)		
2–3 times/week	43 (39.1)	69 (42.9)		
Once/day	7 (6.4)	8 (5.0)		
>Once/day	2 (1.8)	1 (0.6)		
**Fish**			5.8923	0.208
Never	0 (0.0)	1 (0.6)		
Seldom	0 (0.0)	3 (1.9)		
2–3 times/week	26 (23.6)	25 (15.5)		
Once/day	46 (41.8)	65 (40.4)		
>Once/day	38 (34.6)	67 (41.6)		
**Fast Foods** ***			**8.0146**	**0.032**
Never	53 (48.1)	51 (31.7)		
Seldom	48 (43.6)	92 (57.1)		
2–3 times/week	9 (8.2)	17 (10.6)		
Once/day	0 (0.0)	1 (0.6)		
**Coffee**			**10.8889**	**0.025**
Never	24 (21.8)	46 (28.6)		
Seldom	48 (43.6)	57 (35.4)		
2–3 times/week	19 (17.3)	43 (26.7)		
Once/day	19 (17.3)	13 (8.1)		
>Once/day	0 (0.0)	2 (1.2)		
**Salty Foods** ****			**8.2718**	**0.027**
Never	56 (50.9)	61 (37.9)		
Seldom	44 (40.0)	91 (56.5)		
2–3 times/week	9 (8.2)	9 (5.6)		
Once/day	1 (0.9)	0 (0.0)		
**Eggs**			5.3876	0.146#
Never	12 (10.9)	10 (6.2)		
Seldom	31 (28.2)	45 (28.0)		
2–3 times/week	55 (50.0)	97 (60.3)		
Once/day	12 (10.9)	9 (5.6)		
**Peanut butter and nuts**			1.2393	0.746
Never	20 (18.4)	28 (17.4)		
Seldom	55 (50.5)	81 (50.3)		
2–3 times/week	28 (25.7)	47 (29.2)		
Once/day	6 (5.5)	5 (3.1)		
**Dry beans, pea and soya beans**			1.1249	0.916
Never	2 (1.8)	6 3.0)		
Seldom	64 (58.2)	95 (59.4)		
2–3 times/week	39 (35.5)	52 (32.5)		
Once/day	4 (3.6)	5 (3.1)		
>Once/day	1 (0.9)	2 (1.3)		
**Fruits and pure fruit juice**			4.9063	0.346
Never	0 (0.0)	4 (2.5)		
Seldom	23 (20.9)	29 (18.0)		
2–3 times/week	56 (50.9)	77 (47.8)		
Once/day	29 (26.4.0)	43 (26.7)		
>Once/day	2 (1.8)	8 (5.0)		
**Dark green leafy vegetables and others**			4.5189	0.304
Never	1 (0.9)	1 (0.6)		
Seldom	15 (13.6)	19 (11.8)		
2–3 times/week	71 (64.6)	117 (72.7)		
Once/day	21 (19.1)	18 (11.2)		
>Once/day	2 (1.8)	6 (3.7)		
**Tubers, potatoes and cocoyam**			5.1954	0.323
Never	0 (0.00)	2 (1.2)		
Seldom	34 (30.91)	52 (32.3)		
2–3 times/week	69 (62.73)	101(62.7)		
Once/day	7 (6.36)	4 (2.5)		
>Once/day	0 (0.00)	2 (1.2)		
**Bread, cereals, rice and pasta**			3.2699	0.534
Never	1 (0.9)	4 (2.5)		
Seldom	9 (8.2)	7 (4.4)		
2–3 times/week	49 (44.6)	80 (49.7)		
Once/day	46 (41.8)	65 (40.4)		
>Once/day	5 (4.6)	5 (3.1)		

^#^Chi square p-value.

**Sweets comprise foods such a cheese, yoghurts, ice cream, sweets and soft drinks.

***Fast foods comprise foods such as noodles (indomie), KFCs, pizza, etc.

****Salty foods include fried yam, potato and plantain chips, salted tilapia, etc.

Table percentages were arrived at using column totals.

### Effect of comorbidities with T2DM and glycaemic control

The most common comorbidity found among people with T2DM was hypertension (73.43%) and followed by an abnormality in lipid metabolism (dyslipidemia) (16.97%). Common complications associated with uncontrolled T2DM were neuropathy (40%), cardiovascular diseases (32%), diabetic sore (28%), kidney disease (9.23%), retinopathy (25%), spondylosis/musculoskeletal disorder (10%), hypo-sexual arousal (6%), and benign prostate hyperplasia (BPH) (3%). Except for cardiovascular diseases (X^2^: 7.2229, p = 0.007) and diabetic sore (X^2^: 4.7546, p = 0.029) which were found to be statistically significantly associated with glycaemic control, none of the other co-morbidities or complications showed a significant association (Table 5).

**Table 5:** Effects of comorbidities and DM complications on glycaemic control.

Variables	Number (%.)	Good Glycaemic Control (n = 110) (%)	Poor Glycaemic Control (n = 161) (%)	Chi Square	*P*-value
**Hypertension**					
	199 (73.43)	80 (72.73)	119 (73.91)	0.0471	0.828*
**Retinopathy**					
	25 (9.23)	11 (10.00)	14 (8.70)	0.1328	0.716*
**Neuropathy**					
	40 (14.76)	18 (16.36)	22 (13.66)	0.3784	0.538*
**Chronic Kidney Disease**					
	25 (9.23)	11 (10.00)	14 (8.70)	0.1328	0.716*
**Other Cardiovascular Diseases**					
	32 (11.81)	20 (18.18)	12 (7.45)	**7.2229**	**0.007** *
**Diabetic Sores**					
	28 (10.33)	6 (5.45)	22 (13.66)	**4.7546**	**0.029** *
**Hypo -Sexual Arousal**					
	6 (2.21)	4 (3.64)	2 (1.24)	1.7302	0.227#
**Benign Prostate Hyperplasia**					
	3 (1.11)	0 (0.00)	3 (1.86)	2.0726	0.274#
**Dyslipidaemia**					
	46 (16.97)	17 (15.45)	29 (18.01)	0.3034	0.582*
**Spondylosis/Musculoskeletal Disorders**					
	10 (3.69)	5 (4.55)	5 (3.11)	0.3812	0.533#

^#^Fisher’s exact p-value.

*Chi square (X^2^) p-value*.

### Other factors associated with glycaemic control

Other variables that could influence HbA1c test results and thus glycaemic control, were assessed and the results showed that none of the following parameters; aspirin, being on anti-retroviral drugs, erythropoietin, iron, and vitamin B12 supplements; being a smoker; alcohol intake; having splenectomy; recent transfusion; splenomegaly; arthritis; and sickle cell disease—known to affect Glycaemic control or the monitoring test (HbA1c) directly or indirectly—significantly influenced glycaemic control in any way (Table 6).

**Table 6:** Association between HbA1c test interferences and glycaemic control.

Variables	Number (%.)	Good Glycaemic Control (n = 110) (%)	Poor Glycaemic Control (n = 161) (%)	Chi Square statistic	*p*-value (Fishers’ exact)
**Erythropoietin**					
	2 (0.74)	1 (0.93)	1 (0.93)	0.0814	1.000
**Aspirin**					
	13 (4.81)	5 (4.55)	8 (5.00)	0.0294	1.000
**Iron or Vitamin B12 Supplements**					
	8 (2.95)	1 (0.91)	7 (4.35)	2.6974	0.148
**Anti-Retroviral Drugs**					
	2 (0.74)	2 (1.82)	0 (0.00)	2.9126	0.166
**Smoking**					
	11 (4.06)	5 (4.55)	6 (3.73)	0.1125	0.737*
**Alcohol Intake**					
	78 (28.78)	30 (27.27)	48 (29.81)	0.2058	0.650*
**Splenectomy**					
	1 (0.37)	1 (0.91)	0 (0.00)	1.4691	0.406
**Recent Transfusion**					
	8 (2.95)	3 (2.73)	5 (3.11)	0.0326	1.000
**Sickle Cell Disease**					
	3 (1.11)	2 (1.82)	1 (0.62)	0.8554	0.568
**Arthritis/Splenomegaly**					
	9 (3.32)	6 (5.45)	3 (1.86)	2.6250	0.165

* Chi square (X^2^) p-value.

### Patient perception of the quality of health service delivery and glycaemic control

Study participants were asked of their perception of being involved in the T2DM treatment, respect and privacy accorded them during clinic visits, the time of delay (waiting time) at the facility, and their view on the availability of space, adequate health professionals and machines/instruments, among others. None of the respondents’ views on the various aspects of health service delivery was found to be statistically significantly associated with glycaemic control.

### Association of key variables and glycaemic control

Bivariate logistic regression was performed to determine the strength of association of the variables that showed a significant effect on glycaemic control from the Chi-square tests to obtain crude odds ratios (cOR) (Table 7). Further, together with theoretically known variables that affect HbA1c, multiple logistic regression was conducted to obtain the adjusted odds ratios (AOR). From the analysis, having low education (only elementary or JHS), being morbidly obese, being on insulin therapy, having cardiovascular disease, and taking coffee seldom or once per day significantly affected one’s glycaemic control. Whereas the intake of coffee and having CVD showed protective action in T2DM (reduction of HbA1c), there was an increase in glycosylated haemoglobin (poor glycaemic control) with attaining only elementary education, being morbidly obese or being on insulin therapy. For instance, there were approximately three-times increased odds of having poor glycaemic control if one one was on insulin therapy compared to not being on the therapy (AOR: 2.88; 95% CI: 1.05 to 7.88; p = 0.04).

**Table 7:** Logistic regression model of key variables and glycaemic control.

Variables	cOR*	p-value	95% C. I.	AOR#	p-value	95% C. I.**
**High Physical Activity (Reference)**						
Moderate Activity	0.78	0.597	0.309–1.965	0.54	0.288	0.170–1.692
Low Activity	1.57	0.306	0.662–3.709	0.92	0.879	0.306–2.750
**Tertiary Level of Education (Reference)**						
SHS Education	1.95	0.058	0.979–3.889	2.94	0.110	0.783–11.031
Elementary/JHS	3.35	**<0.0001**	**1.747–6.407**	5.22	**<0.0001**	**2.120–12.856**
No formal education	2.40	0.077	0.909–6.339	1.95	0.142	0.800–4.777
**Normal BMI (Reference)**						
Overweight	1.32	0.402	0.689–2.531	1.18	0.693	0.512–2.733
Obese	2.08	**0.049**	**1.004–4.321**	1.62	0.307	0.641–4.114
Morbidly Obese	5.00	**0.020**	**1.292–19.356**	**13.22**	**0.008**	**1.946–89.803**
**Insulin Therapy**	2.68	**0.010**	**1.262–5.680**	**2.88**	**0.040**	**1.050–7.881**
**Cardiovascular Disease**	0.36	**0.009**	**0.169–0.777**	**0.15**	**0.001**	**0.049–0.456**
**Intake of Fast Foods (Reference = Never)**						
Seldom	1.99	**0.009**	**1.185–3.348**	1.37	0.388	0.669–2.809
2–3 times/week	1.96	0.140	0.802–4.803	2.36	0.193	0.648–8.580
**Intake of Coffee (Reference = Never)**						
Seldom	0.62	0.133	0.332–1.158	**0.27**	**0.003**	**0.115–0.638**
2–3 times/week	1.18	0.656	0.568–2.454	0.84	0.745	0.319–2.266
Once/day	0.36	**0.019**	**0.151–0.844**	0.19	0.005	0.061–0.609
**Intake of Salty Foods (Reference = Never)**						
Seldom	1.90	**0.014**	**1.139–3.165**	1.69	0.148	0.829–3.467
2–3 times/week	0.92	0.866	0.340–2.477	0.98	0.982	0.258–3.767

*cOR = Crude odds ratio.

^#^AOR = Adjusted odds ratio.

**95% CI = 95% confidence interval.

### Participants’ recommendations for effective treatment of their diabetes

Some participants (28.8%; n = 78) recommended diet control to ensure optimal blood glucose control. Many described this action to be well planned with specialists and dieticians, and hence the suggestion of more doctors, dieticians, etc. by 71 (23.6%) respondents. Notable was the assertion that patients were generally informed of restraining from certain foods categories without specifically being given specific names of these foods and a diet plan to follow.

Some respondents recommended education (17.3%) and physical activity (17.7%) as ways of effectively controlling their blood glucose level. A few (5.9%) recommended self-discipline as an effective way of glycaemic control whereas 7.0% indicated that the medication and some laboratory tests were expensive; they were not listed on Ghana’s National Health Insurance drug lists and hence purchasing them becomes a problem. Others also alluded to difficulty in meeting the financial demands of their homes. They pleaded for financial support from government and non-governmental agencies to help control/manage their condition.

## Discussion

This study assessed various factors that could influence blood glycaemic control among people with T2DM visiting the Greater Accra Regional Hospital, Ghana. Overall, high BMI and use of insulin therapy were associated with poor glycaemic control (high HbA1c) whereas low levels of coffee intake and having a cardiovascular disease were positively associated with glycaemic control. Self-monitoring blood glucose and diet therapy did not appear to influence glycaemic control. When age and gender were controlled, high physical activity was positively associated with blood glucose control.

### Sub-optimal glycaemic control

In our study, majority (69.7%) of participants had HbA1c values ≥7.1% which exceeds the optimal HbA1c target of 7.0% (53.0 mmol/mol) during diabetes mellitus treatment [6, 7]. This has serious implications for public health and clinical practice as it indicates that majority of patients receiving treatment in hospitals are not achieving glycaemic control. Better still, if we assume that less stringent goal of HbA1c value <8.0% (64 mmol/mol) was set by clinicians due to extensive comorbid conditions of patients, due to patients’ history of severe hypoglycemia, and as a results of advanced micro and macro vascular complications of patients as proposed by the ADA (2019) [25] and the fact that laboratory results that clinicians mostly deal with in managing patients with diabetes in Ghana quote ≤8.0% (64mmol/mol) as good control, there is still room for improvement if 59.4% participants recorded HbA1c of 8.1% (65mmol/mol) and above.

### BMI, education, diet therapy and other determinants of blood glucose control

A study by Szkudelski and Szkudelska [26] indicated that T2DM is mostly associated with overweight and obese individuals. Consistent with this finding, our study had majority (80.5%) of the participants being overweight, obese, or morbidly obese. Notably, BMI was found to be strongly associated with glycaemic control among participants in our study. Being morbidly obese increased one’s odds of having poor glycaemic control by five (compared to having normal BMI), after controlling for all essential parameters. Meanwhile, the higher one’s blood glucose, the greater the risk of comorbidities and complications onset [27], hence controlling the BMI can go a long way to improve the quality of life in T2DM affected persons. Additionally, a study by Whiltlock et al [28] showed that being morbidly obese (BMI of 40–45 Kg/m^2^) reduces survival rate by 8–10 years; thus, mortality is directly proportional to BMI.

Meanwhile, diet control is an effective means of controlling one’s BMI, which may ultimately affect individuals’ glycaemic control. However, our study found no significant association between diet and glycaemic control, despite the different categorizations done using recommended dietary ranges.

This finding may be because almost none of the participants in our study was on strict diet therapy. Also, accurately estimating the amount of dietary intake, especially within the Ghanaian context, may have been difficult for many participants and thus, prone to some response bias. Noteworthy, current evidence remains inconclusive on the exact dietary requirements for people with T2DM. While stressing the need for diabetes self-management education (DSME) and registered dietician’s counselling, experts indicate that dietary plans for people with T2DM should be bendable and individualized, but not "one-size-fits-all" dietary sheets [23]. This necessitates the compulsory inclusion of registered dietician’s comprehensive counselling and guidance in the management of T2DM and not restricting this to a limited few.

Patient Education Programme (PED) for people with T2DM is a difficult one but has significant impacts hence it has always been recommended for effective control of blood glucose especially at community health centers [29]. It is this method that has been employed in treatment and management of patients with Type 1 Diabetes in the DAFNE (Dose Adjustment for Normal Eating Educational) programme. The programme aimed at training patients to own their glycaemic control and disease management with specialized skill obtained in a well-structured educational programme; and which had the benefit of reducing the occurrence of hypoglycaemia among its beneficiaries, improving quality of life and giving optimal glycaemic control, as well as improving psychosocial outcomes [30, 31]. DAFNE has been known to work and generate highly positive results in Germany and currently thriving in the UK [19] In this study, having only elementary or Junior High School education significantly increases one’s odds of having poor glycaemic control compared to having tertiary education (AOR = 3.35; 95% CI 2.017–12.469; P < 0.0001). Since there was no significant association with respondents with no formal education, we argue that only a little bit of education and knowledge is required to make a huge impact or change in one’s glycaemic control.

With even respondents recommending education as a means of helping to adequately control their blood glucose, we hypothesize that if patients are well educated concerning their condition, they will tend to adhere more to treatment modalities, and improvement in their condition can be guaranteed. Behavioural feasibility of physical activity and motivation of patient towards treatment has been described to be a great key for improvement in treatment intervention [32] and these are some areas that tend to be of benefit from education.

Several studies suggest that the more one’s blood glucose increases, the higher the risk in developing T2DM-related complications. Selvin et al. [33] identified that for 1% increase in HbA1c, there is an 18% increased risk of developing cardiovascular events, whilst Stratton et al [34]found that the risk of developing diabetic retinopathy or renal failure is increased by 37% for a 1% rise in the HbA1c of individuals with T2DM. As such, considering the relatively high HbA1c of participants in this study, it was not surprising that over half of our study participants each had one to three or more diabetes complications of retinopathy, neuropathy, diabetic foot, hypertension and other CVDs, hypoactive sexual arousal and many others (Table 6). However, none of these except other CVDs (AOR = 0.36; 95% CI 0.169–0.777; P = 0.009) were found to be statistically significant with glycaemic control. A randomized trial in the Action to Control Cardiovascular Risk in Diabetes (ACCORD) study [35] reported that controlling blood pressure of 4,733 participants to obtain normal systolic pressure of <120 mmHg among people with T2DM resulted in no significant change in the rate of a composite outcome of non-fatal and fatal major cardiovascular events but not glycaemic control. Most probably there was a medication being taken by almost all the patients with CVD in this study that affects HbA1c results which was not accounted for, or the condition really had protective action, an area that requires more extensive research. Just like being on insulin therapy was found to be statistically significant with glycaemic control (AOR = 2.68; 95% CI 1.262–5.680; p = 0.01). We argue that this could be a reverse cause effect where those with higher blood glucose are more likely to be put on insulin therapy or very stringent medication therapy till they achieve near normal glycaemic control and to prevent onset of complications before medication therapy is employed or made more flexible [7].

Noteworthy, compared to never taking coffee, our data point out that seldom coffee intake (AOR 0.27, 95% CI 0.115–0.638, p = 0.003) was significantly associated with good glycaemic control (lowering of HbA1c) (Tables 4 and 7). We believe this finding is a co-incidence since we could not find any related literature or scientific explanation to it. Further studies in the area is recommended to throw more light on the topic.

### Effective control of factors affecting glycaemic control

Theoretical variables such as splenectomy, transfusion, splenomegaly, aspirin use or sickle cell disease, use of erythropoietin, iron and B12 supplements among others [6] known to affect HbA1c results were all found to have no significant effect on the test but were however controlled for in this study. The non-significance which may probably be due to the negligible numbers of these cases and their normal distribution among both individuals with good and poor glycaemic control validates the HbA1c test results used in this study.

A study by Acik et al. [36] showed that to experience oral medication effectiveness in patients with T2DM, combining treatment with diet control and physical activity is critical. Their intervention study, which planned and organized a diet and physical activity of participants saw progress in the glycaemic control of study participants. In the current study, we observed that there was no structured PA nor diet plan for patients as effective treatment modality aside being told to include exercise in treatment. Therefore, we recommend combined and well-planned treatment modality alongside similar recommendations by some participants and Acik et al. [36].

Similarly, other studies have found the use of self-monitoring blood glucose (SMBG) by people with T2DM to reduce their HbA1c, ensure hypoglycemic episodes’ visualization, to lead to glycaemic variability and lifestyle improvement [37–39]. SMBG is a way of randomly checking ones blood glucose irrespective of location with finger pricks and the use of glucometer in most cases to reveal, throughout the day, significant patterns of blood glucose[39]. Our study however did not find any significant association between the SMBG and glycaemic control. We found that only slightly over half of our participants (53.4%) had glucometers and ever used them to check their blood glucose. Possessing a glucometer did not translate into their use for SMBG. Not utilizing personal glucometers was as a results of not having tests strips or lack the technical expertise to operate it. In a study by Danne et al. [40] who recommended testing regimens should be structured for those using SMBG, the method was found to be effective among those on insulin injections as well as those on medications except that the use sorely depended on patients’ interest, and that a single test did not determine the rate of change or change direction in blood glucose control.

### Study limitations and strengths

This study explored the physical activity level of participants as well as their food intake frequency and dietary recall. These results may be prone to recall bias. Additionally, the IPAQ for measuring physical activity is designed for use among individuals aged 15–69 years. However, it was applied to a few older study participants as well in this study due to a lack of appropriate measuring tools. More so, HbA1c results used in the current study were obtained from about three different laboratories. These laboratories may be using different methods in their HbA1c estimation and may be subject to inter-laboratory differences. Additionally, inadequate information on SMBG could hardly validate the effective use of the method as was intended in the study. We also believe conducting this study as a prospective cohort and a multi-center one instead of a single center many have improved on the finding compared to what was obtained. Notwithstanding, the study was holistic in nature as it investigated almost all parameters affecting one’s glycaemic control without taking any of the factors in isolation.

## Conclusion

Our study found nearly two quarters of people with T2DM had poor glycemic control (HbA1c ≥8.1 (65mmol/mol). The high proportion of patients on treatment showing sub-optimal glycaemic control is not ideal for preventing onset of disease complications and early mortality or for improving quality of life which calls for expedite action.

Our data showed that controlling for age and gender, physical activity was significantly associated with glycaemic control (OR = 1.58; 95% CI: 1.059 to 2.346; P = 0.025). Those who exercised more had a higher tendency of controlling their glucose level compared to those who did not engage in physical activity. Low level of education and high BMI poorly affected glycaemic control whiles being on insulin therapy and having a cardiovascular disease were also found to be positively associated with one’s glycaemic control.

Our study results showing a high proportion of patients attending the Diabetes Clinic with uncontrolled diabetes has serious implications on the management of T2DM diabetes and this suggests that current hospital-based treatment measures are less effective. Comprehensive management of T2DM targeting all the key factors identified in this study and incorporating a multispectral collaborative effort based on holistic approach, and combined with non-pharmacological component are strongly warranted. On the question of effective interventions for T2DM, our study respondents recommended practical dietary guidance, education, more health specialists and financial support, among others, as key for effective glycaemic control.

## Supporting information

S1 Fig Flowchart summarizing participants enrolment, eligibility and number included in study analyses. (TIF)

S1 File Study questionnaire, determinants of blood glucose control. (DOCX)

S2 File Complete data set—Blood glucose control, Djonor et al. (XLSX)

S3 File Code sheet—Blood glucose control, Djonor et al. (DOC)

S1 Checklist STROBE 2007 (v4) statement—Checklist of items that should be included in reports of *cross-sectional studies*. (DOCX)
