# Supplementary figures and images for: Determinants of blood glucose control among people with Type 2 diabetes in a regional hospital in Ghana
Source: PLoS One. 2021 Dec 22;16(12):e0261455. doi: 10.1371/journal.pone.0261455 (PMC8694475; doi:10.1371/journal.pone.0261455)

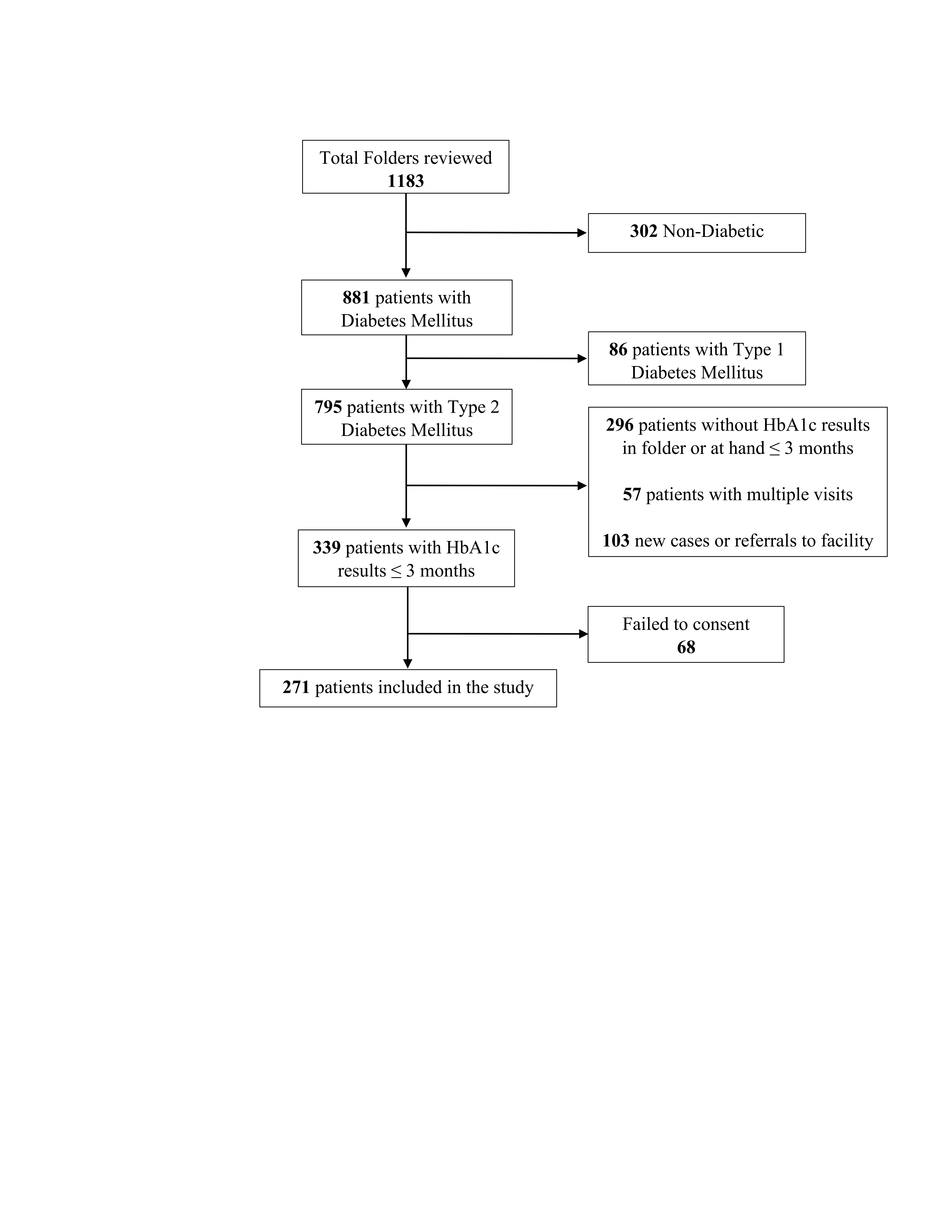

Supplement: S1 Fig — (TIF) [file pone.0261455.s001.tif]
