# Supplementary material for: Determinants of blood glucose control among people with Type 2 diabetes in a regional hospital in Ghana
Source: PLoS One. 2021 Dec 22;16(12):e0261455. doi: 10.1371/journal.pone.0261455 (PMC8694475; doi:10.1371/journal.pone.0261455)
Supplement: S1 File — (DOCX) [file pone.0261455.s002.docx]

## APPENDIX II: STUDY QUESTIONNAIRE

**Determinants of Blood Glucose Control among People with Type 2 Diabetes in a Regional Hospital in Ghana**

Sampson Kafui Djonor ^1,5^, Ignatius Terence Ako-Nnubeng ^1^, Ewurama Ampadu Owusu ^2^, Kwadwo Owusu Akuffo ^3^, Pricillia Nortey ^1^, Eldad Agyei-Manu ^3^, Anthony Danso-Appiah ^1, 4^ *

**SOCIO-DEMOGRAPHIC CHARACTERISTICS AND BASIC DISEASE INFO (PART 1**)

/ /

0.1. Date: 3.0. Religion

/ /

0.2. Interviewer: 3.1. Age (Years):

1.1. Participant ID: 4.1. Gender (M/F)

1.2. Contact No: 5.1. Place of Residence

| 6. What is your Level of education? | | 1. No formal education  2. Elementary  3. JHS | | 4. SHS/Secondary  5. Tertiary education |
| --- | --- | --- | --- | --- |
| 7. What is your occupation group? | | 1. Managers  2. Professionals/skilled  3. Clerical support workers  4. Service and Sales. | | 5. Skilled Agricultural workers  6. Crafts & Related Trade  7. Elementary Occ/Labourer  8. Unemployed |
| 8. What is your estimated monthly level of income? | | 1. Ghȼ199.00 or less  2. Ghȼ200.00 - Ghȼ499.00  3. Ghȼ500.00 - Ghȼ999.00 | | 4. Ghȼ1,000.00 - Ghȼ1,999.00  5. Ghȼ2,000.00 - Ghȼ3,999.00  6. Ghȼ4,000.00 & Above |
| 9. How long have you been of diabetes treatment? | | 1. Less than a year  2. Two to five years | | 3. Five to nine years  4. Ten to 19years  5. 20 years and more. |
| 10. What treatment modality are you on? **(Tick as many that apply)** | | 1. Diet  2. Physical Activity | | 3. Insulin therapy (Injection)  4. Oral Medication |
| 11. Which of these complications or conditions do you have?  **(Tick as many that apply)** | | 1. Hypertension (High BP)  2. Retinopathy (Eye disease)  3. Neuropathy (Nerve disease)  4. Cardiovascular disease | | 5. Chronic Kidney Disease  6. Diabetic Sores  7. Hypoactive sexual arousal  8. Other (s):………………………. |
| 12. Which of these complications or conditions are you on treatment for?  **(Tick as many that apply)** | | 1. Hypertension (High BP)  2. Retinopathy (eye disease)  3. Neuropathy (Nerve disease)  4. Cardiovascular disease | | 5. Chronic Kidney Disease  6. Diabetic Sores  7. Hypoactive sexual arousal  8. Other (s):………………………. |
| 13. How do you rate timely service (the waiting time of care) at the facility? | | 1. Good  2. Fair | | 3. Poor |
| 14. How have you been involved in the treatment of the condition?  (Education; Motivation; Treatment explanation; Opinions, etc.) | | 1. Very much involved  2. Fairly involved | | 3. Poorly Involved |
| 15. How do you rate respect and privacy accorded you by healthcare professionals? | | 1. Good  2. Fair | | 3. Poor |
| 16. What is your idea on your improvement status? | | 1. Condition has Improved  2. No change in status | | 3. Worsened |
| 17. Who is associated with your improved, no change or worsened state of condition? | 1. Myself  2. Health Professionals | | 3. Family Members  4. I have no idea | |
| 18. Rate availability of care instruments, facilities (for space, BP check, lab, etc.) and human resources to cater for your health | 1. Good  2. Fair | | 3. Poor | |
| 19. Which of the following drugs are you on? **(Tick as many that apply)** | 1. Erythropoietin  2. Aspirin  3. Food supplements (Iron, B12) | | 4. Anti -Retroviral Drugs  5. Other(s)…………………………  …………………………………… | |
| 20. Do you Smoke? | 1. Yes | | 2. No | |
| 21. How often do you take in alcohol? | 1. Every Day  2. Less than 3 times a week | | 3. Few times in a month/year  4. Not at all | |
| 22. Which of the following applies to you? | 1. Had splenectomy in surgery  2. Recently received transfusion  3. Have Sickle cell/thalassemia | | 4. Have splenomegaly/Arthritis  5. None | |
| 23. What are your recommendations for effective treatment? | 1.  2. | | 3.  4. | |
| *24. What is participant’s HbA1c results?* | *1. Within last 3months?*  *……….% or………….mmol/mol* | | *2. Within 1^st^ year of treatment or diagnosis? …...% or …....mmol/mol* | |
| *25. What is participant’s BMI results?* | *1. Within last 3months?*  *…………………. Kg/m^2^* | | | |
| 26. Do you have Glucometer at home for self-monitoring? | Yes | | No | |
| 27. Why don’t you have or use Glucometer at home for self-monitoring? (Tick as many that apply) | 1. Money to buy strips/machine  2. I know not of self-monitoring  3. Testing/Interpretation problem | | 4. I just do not want to  5. It is not my duty  6. Other:…………………………. | |

**ASSESSMENT OF PHYSICAL ACTIVITY (PART 3)**

| Next I am going to ask you about the time you spend doing different types of physical activity in a typical week. Please answer these questions even if you do not consider yourself to be a physically active person. Think first about the time you spend doing work. Think of work as the things that you have to do such as paid or unpaid work, study/training, household chores, harvesting food/crops, fishing or hunting for food, seeking employment.  In answering the following questions: **'vigorous-intensity activities'** are activities that require hard physical effort and cause large increases in breathing or heart rate; **'moderate-intensity activities'** are activities that require moderate physical effort and cause small increases in breathing or heart rate. | | |
| --- | --- | --- |
| **QUESTION** | **RESPONSE** | **CODE** |
| **Work** | | |
| Does your work involve vigorous-intensity activity that causes large increases in breathing or heart rate like [carrying or lifting heavy loads, digging or construction work] for at least 10 minutes continuously? | Yes 1  No 2  **If No, go to P4** | **P1** |
| In a typical week, on how many days do you do vigorous intensity activities as part of your work? | Number of days | **P2** |
| How much time do you spend doing vigorous-intensity activities at work on a typical day? | Hours : minutes   \|  \|  \| : \|  \|  \| \| --- \| --- \| --- \| --- \| --- \| | **P3 (a-b)** |
| Does your work involve moderate-intensity activity that causes small increases in breathing or heart rate such as brisk walking [or carrying light loads] for at least 10 minutes continuously? | Yes 1  No 2  **If No, go to P7** | **P4** |
| In a typical week, on how many days do you do moderate-intensity activities as part of your work? | Number of days | **P5** |
| How much time do you spend doing moderate-intensity activities at work on a typical day? | Hours : minutes   \|  \|  \| : \|  \|  \| \| --- \| --- \| --- \| --- \| --- \| | **P6 (a-b)** |
| **Travel to and from Places** |  |  |
| *The next questions exclude the physical activities at work that you have already mentioned. Now I would like to ask you about the usual way you travel to and from places. For example to work, for shopping, to market, to place of worship*. | | |
| Do you walk or use a bicycle (pedal cycle) for at least 10 minutes continuously to get to and from places? | Yes 1  No 2  **If No, go to P10** | **P7** |
| In a typical week, on how many days do you walk or bicycle for at least 10 minutes continuously to get to and from places? | Number of days | **P8** |
| How much time do you spend walking or bicycling for travel on a typical day? | Hours : minutes   \|  \|  \| : \|  \|  \| \| --- \| --- \| --- \| --- \| --- \| | **P9 (a-b)** |

| **Physical Activity Assessment; Continued** |  |  |
| --- | --- | --- |
| **QUESTION** | **RESPONSE** | **CODE** |
| **Recreational Activities** |  |  |
| *The next questions exclude the work and transport activities that you have already mentioned. Now I would like to ask you about sports, fitness and recreational activities (leisure)* | | |
| Do you do any vigorous-intensity sports, fitness or recreational (leisure) activities that cause large increases in breathing or heart rate like [running or football] for at least 10 minutes continuously? | Yes 1  No 2  **If No, go to P13** | **P10** |
| In a typical week, on how many days do you do vigorous-intensity sports, fitness or recreational (leisure) activities? | Number of days | **P11** |
| How much time do you spend doing vigorous-intensity sports, fitness or recreational activities on a typical day? | Hours : minutes   \|  \|  \| : \|  \|  \| \| --- \| --- \| --- \| --- \| --- \| | **P12 (a-b)** |
| Do you do any moderate-intensity sports, fitness or recreational (leisure) activities that cause a small increase in breathing or heart rate such as brisk walking, [cycling, swimming, volley ball, etc.] for at least 10 minutes continuously? | Yes 1  No 2  **If No, go to P16** | **P13** |
| In a typical week, on how many days do you do moderate-intensity sports, fitness or recreational (leisure) activities? | Number of days | **P14** |
| How much time do you spend doing moderate-intensity sports, fitness or recreational (leisure) activities on a typical day? | Hours: minutes   \|  \|  \| : \|  \|  \| \| --- \| --- \| --- \| --- \| --- \| | **P15 (a-b)** |
| **Sedentary Behaviour** | | |
| *The following question is about sitting or reclining at work, at home, getting to and from places, or with friends including time spent sitting at a desk, sitting with friends, traveling in car, bus, train, reading, playing cards or watching television, but do not include time spent sleeping.* | | |
| How much time do you usually spend sitting or reclining on a typical day? | Hours : minutes   \|  \|  \| : \|  \|  \| \| --- \| --- \| --- \| --- \| --- \| | **P16 (a-b)** |

**DIETARY ASSESSMENT (PART 4 & 5)**

**24-HOUR DIETARY RECALL (PART 4)**

I would like to know what you’ve eaten within the past 24 hours. Please tell me everything you ate or drank, including meals, snacks, beverages, candy and alcohol? Why don’t you start with the last thing you’ve had to eat or drink today and we’ll go backwards.

|  | | | | Dairy  Products | Grains | Meat or  Substitute | Fruits | Vegetables | Fats, Oils Sweets |
| --- | --- | --- | --- | --- | --- | --- | --- | --- | --- |
| Time | Place | Food or Beverage | Amount | For Official Use Only | | | | | |
|  |  |  |  |  |  |  |  |  |  |
|  |  |  |  |  |  |  |  |  |  |
| Recommended servings/day | | |  |  |  |  |  |  |  |
| Is this a typical normal day? ___________________  Total Number of Servings | | |  |  |  |  |  |  |  |

**For Office Use Only Please**

Nutrients diet may be lacking in: ___________________

Nutrients diet may be excessive in: ___________________

**FOOD FREQUENCY QUESTIONNAIRE (PART 5)**

***How often do you eat the following foods? (Tick in the box that applies to you.)***

|  | **More than**  **once/day** | **Once/day** | **2-3 times/week** | **Seldom** | **Never** |
| --- | --- | --- | --- | --- | --- |
| Milk |  |  |  |  |  |
| Cheese, yogurt, Ice cream |  |  |  |  |  |
| Meat, Poultry |  |  |  |  |  |
| Fish |  |  |  |  |  |
| Eggs |  |  |  |  |  |
| Peanut butter, nuts |  |  |  |  |  |
| Dry beans, peas, soya beans |  |  |  |  |  |
| Fruits, juice (i.e. orange, grapefruit, banana, pineapple, watermelon, tangerine, pawpaw, etc.) |  |  |  |  |  |
| Dark green leafy vegetables and others (i.e., collards, broccoli, carrots, squash, sweet potatoes) |  |  |  |  |  |
| Tubers, potatoes, cocoyam, etc. |  |  |  |  |  |
| Bread, cereals, rice, pasta |  |  |  |  |  |
| Sweets (cakes, donuts, pies, cookies, candy) |  |  |  |  |  |
| Salty snacks: potato chips, corn chips, tortilla chips, pretzels, etc. |  |  |  |  |  |
| Alcohol (beer, Guinness, star, wine, etc.) |  |  |  |  |  |
| Coffee, tea |  |  |  |  |  |
| Vitamins, herbs, other supplements |  |  |  |  |  |
| Fast foods (indomie/noddles, KFC, Pizza, etc.) |  |  |  |  |  |
| Soft Drinks (Coca Cola, Fanta, Alvaro, Malt, etc.) |  |  |  |  |  |

**The end.**

**Thank you for your time and efforts.**
