# Supplementary material for: Determinants of blood glucose control among people with Type 2 diabetes in a regional hospital in Ghana
Source: PLoS One. 2021 Dec 22;16(12):e0261455. doi: 10.1371/journal.pone.0261455 (PMC8694475; doi:10.1371/journal.pone.0261455)
Supplement: S3 File — (DOC) [file pone.0261455.s004.doc]

**CODE SHEET: BLOOD GLUCOSE CONTROL – SAMPSON KAFUI DJONOR**

|  | **VARIABLE DESCRIPTION** | **VARIABLE NAME** | **VALUE LABELS** |
| --- | --- | --- | --- |
|  | Identification | id |  |
|  | Age | age | …….. |
|  | Gender | gender | [1]Male  [2] Female |
|  | Religion | Religion | [1] Christianity  [1] Islam  [2] Traditional  [3] Other |
|  | Residence | Resid | [1] Urban  [2] Peri-urban  [3] Rural |
|  | Education | Educ | [0] Tertiary educ  [1] No formal educ  [2] Elementary/JHS  [3] SHS educ |
|  | Occupation | Occup | [1] Managers  [2] Professional/Skilled  [3] Clerical Support  [4] Service and Sales  [5] Agricultural  [6] Craft & Related Trade  [7] Elem. Occ./Labourer  [8] Unemployed |
|  | Income | Income | [1] < Ghȼ200  [2] Ghȼ200 - Ghȼ499  [3] Ghȼ500 - Ghȼ999  [4] Ghȼ1,000 - Ghȼ1,999  [5] Ghȼ2,000 - Ghȼ3,999  [6] Ghȼ4,000 and Over |
|  | Duration of diabetes condition | Duration | [1] Within a year  [2] 2 – 5 years  [3] 5 -10 years  [4] 10 – 20 years  [5] 20 years and Above |
|  | On diet therapy | Diet | [0] No  [1] Yes |
|  | On exercise therapy | Exerct | [0] No  [1] Yes |
|  | On insulin therapy | Insulint | [0] No  [1] Yes |
|  | On medication therapy | Medt | [0] No  [1] Yes |
|  | Has hypertension | Hptn | [0] No  [1] Yes |
|  | Has retinopathy | retinopathy | [0] No  [1] Yes |
|  | Has Neuropathy | Neuropathy | [0] No  [1] Yes |
|  | Has cardiovascular disease | Cvd | [0] No  [1] Yes |
|  | Has diabetic sores | Sores | [0] No  [1] Yes |
|  | Has hyposexual arousal | Hsar | [0] No  [1] Yes |
|  | Has benigh prostate hyperplasia | Bph | [0] No  [1] Yes |
|  | Has dyslipidemia | Dyslipid | [0] No  [1] Yes |
|  | Has spondylosis | Spondylosis | [0] No  [1] Yes |
|  | Waiting time at the facility | Wait | [0] Good  [1] Fair  [2] Poor |
|  | Patients thought of being involved in treatment | Involve | [0] Good  [1] Fair  [2] Poor |
|  | Respect given to patients during hospital visitations | Respect | [0] Good  [1] Fair  [2] Poor |
|  | Patients idea on improvement status | improve | [1] Improved  [2] No change  [3] Worsened |
|  | Attributes improvement to – myself | myself | [0] No  [1] Yes |
|  | Attributes improvement to – healthcare workers | hcw | [0] No  [1] Yes |
|  | Attributes improvement to – family members | family | [0] No  [1] Yes |
|  | Has no idea who to – Attributes improvement to | No_idea | [0] No  [1] Yes |
|  | Attributes improvement to – God | God | [0] No  [1] Yes |
|  | Availability of Space, HCW, Machines | availability | [0] Good  [1] Fair  [2] Poor |
|  | On Erythropoeitin | Erythrop | [0] No  [1] Yes |
|  | On Aspirin | Aspirin | [0] No  [1] Yes |
|  | On Iron supplements | Iron | [0] No  [1] Yes |
|  | On antiretroviral therapy | Arvs | [0] No  [1] Yes |
|  | Smoke? | Smoke | [0] No  [1] Yes |
|  | Take in alcohol? | Alcohol1 | [0] No  [1] Yes |
|  | Had splenectomy? | Splenectomy | [0] No  [1] Yes |
|  | Had blood transfusion in the last 3 months? | transfusion | [0] No  [1] Yes |
|  | Has sickle cell disease? | Scd | [0] No  [1] Yes |
|  | Diagnosed of splenomegaly? | Splenomegaly | [0] No  [1] Yes |
|  | Patient recommends exercise therapy as DM control means | Rec_exerc | [0] No  [1] Yes |
|  | Patient recommends education as DM control means | Rec_educ | [0] No  [1] Yes |
|  | Patient recommends diet therapy as DM control means | Rec_diet | [0] No  [1] Yes |
|  | Patient recommends medication adherence as DM control means | Rec_adhere | [0] No  [1] Yes |
|  | Patient recommends specialists as DM control means | Rec_spec | [0] No  [1] Yes |
|  | Patient recommends financial support as DM control means | Rec_support | [0] No  [1] Yes |
|  | Patient recommends self-discipline as DM control means | Rec_selfdisc | [0] No  [1] Yes |
|  | Current HbA1c value | Hba1c |  |
|  | HbA1c value at baseline treatment (earlier HbA1c) | Ehba1c |  |
|  | Latest BMI (Kg/m^2^) | bmi |  |
|  | Use of self-monitoring blood glucose | Smbg | [0] No  [1] Yes |
|  | Reason for non-use of self-monitoring blood glucose | Nsmbg | [1] Financial constraints  [2] No idea of SMBG  [3] Testing and result interpretation challenge  [4] No idea/reason |
|  | Level of physical activity | Pactivity | [0] High PA  [1] Moderate PA  [2] Low PA |
|  | Frequency of intake of dairy products | dairyp |  |
|  | Frequency of intake of icecreams | ice |  |
|  | Frequency of intake of meat | meat |  |
|  | Frequency of intake of fish | fish |  |
|  | Frequency of intake of eggs | eggs |  |
|  | Frequency of intake of butter | butter |  |
|  | Frequency of intake of beans | beans |  |
|  | Frequency of intake of fruits | fruits |  |
|  | Frequency of intake of vegetables | vegs |  |
|  | Frequency of intake of tubers | tubers |  |
|  | Frequency of intake of bread and cereals | breadcereals |  |
|  | Frequency of intake of sugar sweetened beverages | sweets |  |
|  | Frequency of intake of salty snacks | Saltysnacks |  |
|  | Frequency of intake of alcohol | Alcohol2 |  |
|  | Frequency of intake of coffee | coffee |  |
|  | Frequency of intake of herbs | herbs |  |
|  | Frequency of intake of fast foods | Fastfoods |  |
|  | Frequency of intake of softdrinks | Softdrinks |  |
|  |  | Ghba1c |  |
|  | Age categories | gage | [1] 15-24 years  [2] 25-29 years  [3] 30-39 years  [4] 40-49 years  [5] 50-59 years  [6] 60-69 years  [7] 70 years & Above |
|  | BMI categories | bmicat | [1] 18-24.9 Kg/m^2^  [2] 25.0-29.9 Kg/m^2^  [3] 30.0-34.9 Kg/m^2^  [4] 35.0 Kg/m^2^ & Above |
|  |  | Shba1c |  |
|  |  | apactivity |  |
|  |  | Bhba1c | 7.0% or less = 0  Above 7.0 = 1 |
|  |  | Chba1c | 8.0% or less = 0  Above 8.0% = 1 |
| **FOOD ANALYSIS** | | | |
|  | *Total energy in kcal obtained from 24hr dietary recall extraction* | *energykcal* |  |
|  | Amount of carbohydrates in grams obtained from 24hr dietary recall extraction | Carbohydrateg |  |
|  | Amount of fats in grams obtained from 24hr dietary recall extraction | Fatg |  |
|  | Amount of proteins in grams obtained from 24hr dietary recall extraction | proteing |  |
|  | Amount of fiber in grams obtained from 24hr dietary recall extraction | fibreg |  |
|  | Amount of carbohydrates in kcal | Carbo_kcal |  |
|  | Amount of fats in kcal | Fat_kcal |  |
|  | Amount of proteins in kcal | Protein_kcal |  |
|  | Amount of fibres in kcal | Fibre_kcal |  |
|  | Corrected total energy in kcal (sum of kcals of carbohydrates, fats and proteins)  * Fibre is regarded as part of carbohydrates | *Total_energy_kcal* |  |
|  | Percentage of carbohydrate in diet based on corrected total energy | Percentrage_carbo |  |
|  | Percentage of fat in diet based on corrected total energy | Percentage_fat |  |
|  | Percentage of protein in diet based on corrected total energy | Percentage_protein |  |
|  | Percentage of fibre in diet based on corrected total energy | Percentage_fibre |  |
|  | Categories of carbohydrate consumed in percentage | Percent_carbo_cat | 1 = <40%  2= 40.00 – 60.00%  3 = Above 60.0% |
|  | Additional Categorization of carbohydrate consumed in percentage | Mean_carbo_percent_cat | 0 = Less than mean 63.75798  1 = Greater than mean 63.75798 |
|  | Categories of protein consumed in percentage | Percent_protein_cat | 1 = <10%  2= 10.01 – 20.00%  3 = Above 20.0% |
|  | Additional Categorization of protein consumed in percentage | Mean_protein_percent_cat | 0 = Less than mean 15.79225  1 = Greater than mean 15.79225 |
|  | Categories of fat consumed in percentage | Percent_fat_cat | 1 = <20%  2= 20.01 – 35.00%  3 = Above 35.00% |
|  | Additional Categorization of fat consumed in percentage | Mean_fat_percent_cat | 0 = Less than mean 20.44976  1 = Greater than mean 20.44976 |
|  | Recommended fat composition | Fat_recommended_cat | 0 = Less than mean 30.00  1 = Greater than mean 30.001 |
|  | Categories of fiber consumed in grams per kcal | fiber_cat | 1 = < or = 20g  2= 20.01 – 35.00g  3 = Above 35.00g |
|  | Additional Categorization of fiber consumed by mean | Mean_fiber_cat | 0 = Less than mean 28.45076  1 = Greater than mean 28.45076 |
